# Publisher Statement on “Questioning the HIV-AIDS Hypothesis: 30 Years of Dissent”

**DOI:** 10.3389/fpubh.2015.00037

**Published:** 2015-02-13

**Authors:** Frontiers Editorial Office

**Affiliations:** ^1^Frontiers, Lausanne, Switzerland

**Keywords:** HIV, AIDS, publisher statement, statement of concern, public health

A Statement of Concern was issued by Frontiers about the article “Questioning the HIV-AIDS hypothesis: 30 years of dissent” on 26 September 2014. The following statement summarizes the outcome of our investigation and replaces the Statement of Concern.

Frontiers has received several complaints from public health professionals related to the article “Questioning the HIV-AIDS hypothesis: 30 years of dissent,” which questions the link between HIV and AIDS. Acknowledging the gravity of these concerns, and the implications that the weakening of the HIV-AIDS link has on public health in general, an internal investigation was conducted.

During the course of the investigation, Frontiers has sought expert input from the Specialty Chief Editors of the HIV and AIDS section of *Frontiers in Public Health* and *Frontiers in Immunology*. Based on the conclusion of the investigation the article type of “Questioning the HIV-AIDS hypothesis: 30 years of dissent” has been changed to an Opinion article, which represents the viewpoint of an individual. In addition, a commentary on the article has been published “Commentary on ‘Questioning the HIV-AIDS hypothesis: 30 years of dissent’,” which discusses the concerns and analyzes the viewpoint within a scientific discourse on the topic.

## Conflict of Interest Statement

The author declares that the research was conducted in the absence of any commercial or financial relationships that could be construed as a potential conflict of interest.

